# Hyperinsulinemia Down-Regulates TLR4 Expression in the Mammalian Heart

**DOI:** 10.3389/fendo.2014.00120

**Published:** 2014-07-22

**Authors:** Melody A. de Laat, Kaylynn J. Gruntmeir, Christopher C. Pollitt, Catherine M. McGowan, Martin N. Sillence, Véronique A. Lacombe

**Affiliations:** ^1^Department of Physiological Sciences, Center for Veterinary Health Sciences, Oklahoma State University, Stillwater, OK, USA; ^2^Australian Equine Laminitis Research Unit, School of Veterinary Science, The University of Queensland, Gatton, QLD, Australia; ^3^Institute of Ageing and Chronic Disease, Faculty of Health and Life Sciences, University of Liverpool, Neston, UK; ^4^Earth, Environmental and Biological Sciences, Queensland University of Technology, Brisbane, QLD, Australia

**Keywords:** toll-like receptor, insulin, myocardium, cytokine, clamp technique

## Abstract

Toll-like receptors (TLR) are key regulators of innate immune and inflammatory responses and their activation is linked to impaired glucose metabolism during metabolic disease. Determination of whether TLR4 signaling can be activated in the heart by insulin may shed light on the pathogenesis of diabetic cardiomyopathy, a process that is often complicated by obesity and insulin resistance. The aim of the current study was to determine if supraphysiological insulin concentrations alter the expression of TLR4, markers of TLR4 signaling and glucose transporters (GLUTs) in the heart. Firstly, the effect of insulin on TLR4 protein expression was investigated *in vitro* in isolated rat cardiac myocytes. Secondly, protein expression of TLR4, the pro-inflammatory cytokines interleukin-6 (IL-6) and tumor necrosis factor-alpha (TNF-α) suppressor of cytokine signaling 3 (SOCS3) and GLUTs (1, 4, 8, 12) were examined in the equine ventricular myocardium following a prolonged, euglycemic, hyperinsulinemic clamp. Down-regulation of TLR4 protein content in rat cardiac myocytes was observed after incubation with a supraphysiologic concentration of insulin as well as in the equine myocardium after prolonged insulin infusion. Further, cardiac TLR4 expression was negatively correlated with serum insulin concentration. Markers of cardiac TLR4 signaling and GLUT expression were not affected by hyperinsulinemia and concomitant TLR4 down-regulation. Since TLRs are major determinants of the inflammatory response, our findings suggest that insulin infusion exerts an anti-inflammatory effect in the hearts of non-obese individuals. Understanding the regulation of cardiac TLR4 signaling during metabolic dysfunction will facilitate improved management of cardiac sequela to metabolic syndrome and diabetes.

## Introduction

Metabolic diseases, which include insulin resistance (IR) and metabolic syndrome, are at the forefront of public health concern due to the global obesity epidemic (1, 2). Importantly, IR can persist for many years in human patients before pancreatic decompensation results in the development of diabetes. However, the early pathophysiological mechanisms underlying the long latency period preceding overt diabetes are largely undefined (3). Termed metabolic syndrome, the disease is characterized by hyperinsulinemia and occurs in both humans and animals (4). Interestingly, horses often develop a variant of metabolic syndrome (called equine metabolic syndrome), which is primarily characterized by IR that can persist for years. Similar to humans and small animals, equine metabolic syndrome is also increasingly diagnosed in current times, with equine obesity and IR burgeoning thanks to concentrate-rich diets and reduced levels of exercise (4, 5). Considering that horses with IR exhibit similar features to metabolic diseases in other species, including humans and small animals, comparative metabolism studies in both small and large animals may aid in further elucidating the pathophysiology of metabolic dysfunction across species. Importantly, the recent development of a prolonged, euglycemic, hyperinsulinemic clamp in healthy, adult, disease free horses that are not prone to cardiovascular disease or diabetes mellitus has resulted in an unprecedented opportunity to study the effect of prolonged hyperinsulinemia in this unique large animal model.

The inability of cells to respond appropriately to insulin, which stimulates glucose uptake into the cell, is the hallmark of IR (6). Glucose uptake is tightly regulated by facilitated diffusion and is mediated primarily by a family of membrane proteins called glucose transporters (GLUTs). Reduced glucose uptake not only results in hyperglycemia, which feeds back to stimulate further insulin release by the pancreas thereby creating or exacerbating hyperinsulinemia, but also leads to cellular and target organ dysfunction. Sequela to defective glucose metabolism are leading causes of morbidity and include cardiovascular disease, impaired wound healing, retinal degeneration, and renal failure (7–9). Impaired glucose metabolism and chronic inflammation in the heart leads to diabetic cardiomyopathy, which is a common and serious consequence of uncontrolled metabolic dysfunction (10).

The activation of toll-like receptor (TLR) signaling is essential for innate immune system regulation and results in up-regulation of inflammatory pathways and the release of inflammatory cytokines, such as interleukin-6 (IL-6) and tumor necrosis factor-alpha (TNF-α) (11). However, the role of TLRs in non-immune cells such as the cardiac myocyte, to which inflammation is harmful, is not well defined. Two TLR isoforms (2 and 4) have been identified at the myocyte surface and have been implicated in ischemic cardiac injury and reduced cardiac myocyte survival (12). Although their potential for playing a pathogenic role in diabetic cardiomyopathy is not well evaluated, recent studies have suggested that TLR signaling is linked to impaired glucose metabolism by altering the regulation of glucose transport (11).

Lipopolysaccharide is the classical ligand for TLR4, although free fatty acids (FFAs) are also thought to be key activators of TLR signaling during metabolic disease (13). More recently, studies on TLR4 activation during type 1 diabetes have indicated that other host-derived molecules are capable of regulating TLR4 function (14–16). Importantly, a recent study showed that short-term insulin infusions administered to type 1 diabetic patients decreased TLR expression in mononuclear cells (14). However, similar regulation of TLR signaling in non-immune cells has not been investigated. Determining if insulin is able to modulate TLR4 signaling in the heart may lead to identification of novel mechanisms of metabolic inflammatory disease as well as novel therapeutic targets.

The aims of the current study were to: (1) determine the effect of hyperinsulinemia using both *in vitro* and *in vivo* approaches, on cardiac TLR4 expression in non-obese animals, and (2) investigate the impact of altered cardiac TLR4 expression during hyperinsulinemia on markers of TLR4 signaling (IL-6, TNF-α, and SOCS3) and cardiac GLUT (1, 4, 8, 12) expression. To this end, we used an integrative physiological approach, including an *in vitro* investigation of small animal myocardial TLR4 responsiveness to insulin, and a well-characterized large animal model that enables examination of non-obese, prolonged hyperinsulinemic states (17, 18).

## Materials and Methods

All described experimental protocols were approved by the Animal Care and Use Committee of Oklahoma State University (VM-12-3) and the Animal Ethics Committee of the University of Queensland (SVS/013/08/RIRDC).

### Cardiac myocyte isolation

Healthy Wistar rats (*n* = 5) were anesthetized with an intraperitoneal injection of pentobarbital sodium (1.2 mL). Once profound anesthesia was achieved, heart extraction preceded immediate cannulation of the aorta. Cardiac myocytes were isolated as previously described (19). Briefly, the heart was perfused in a retrograde manner, using a Langendorff apparatus with Krebs Henseleit (KH) buffer (pH 7.4, 37°C, pH 7.35, and oxygenated with 95% O_2_ and 5% CO_2_), containing (in millimolar): 118 NaCl, 4.7 KCl, 1.2 MgSO_4_, 1.25 CaCl_2_, 1.2 KH_2_PO_4_, 25 NaHCO_3_, 11 glucose. Enzymatic digestion was achieved with collagenase (Worthington, NJ, USA). A minimum yield of 80% live myocytes (rod-shaped cells with sharp margins and clear striations), identified with a light microscope immediately following isolation, was considered acceptable.

Following isolation, live myocytes were incubated in triplicate in KH buffer (with 0.125 mM CaCl_2_) under basal conditions or treated with insulin and/or glucose as follows. Myocytes were incubated with no insulin, 100 or 500 μ IU/mL (physiological or supraphysiological concentration, respectively) of insulin in the presence of physiological (5 mM) or high (25 mM) concentrations of glucose. Following 60 min incubation, myocytes were washed prior to cell solubilization [50 mM Tris-HCl, pH 8.0, 150 mM sodium chloride, 1.0% Igepal CA-630 (NP 40), 0.5% sodium deoxycholate, and 0.1% sodium dodecyl sulfate with a protease inhibitor cocktail] and protein extraction.

### Prolonged, euglycemic, hyperinsulinemic clamp

Prolonged marked hyperinsulinemia was induced by the combined infusion of insulin (constant rate, Humulin-R, 6 m IU/kg BW/min) and glucose (variable rate) for 46 ± 2.3 h to maintain a stable blood glucose concentration of 5 ± 1 mM in a large animal model, as previously described (18, 20). Standardbred horses treated with a prolonged, euglycemic, hyperinsulinemic clamp (p-EHC) were matched (age and weight) with healthy control horses that were administered a balanced electrolyte solution (*n* = 4 per group). Blood samples (10 mL) were collected at the start and again at the end of the clamp to determine blood glucose and serum insulin concentrations. Blood glucose was analyzed using fresh, whole blood with a handheld glucometer (Accu Check-Go, Roche); insulin concentration was determined on serum (stored at −80°C) using a radioimmunoassay (Coat-a-count, Siemens Healthcare Diagnostics, IL, USA). Both techniques have been validated for use in this species (18, 21). At the conclusion of the clamp, animals were humanely euthanized and striated muscles (mid-gluteal muscle and left ventricle) collected. Samples were immediately frozen in liquid N and stored at −80°C until further analysis.

### Protein extraction and western immunoblotting

Crude membrane and total protein was extracted from frozen tissue samples (50 mg) for each horse, and total protein extracted from fresh cardiac myocytes, as previously described (22, 23). Briefly, for crude membrane extraction, tissues were homogenized (BioSpec, OK, USA) in buffer (210 mM sucrose, 40 mM NaCl, 2 mM EGTA, 30 mM Hepes, with a protease inhibitor cocktail). Cells were lysed (1.2 M KCl) prior to ultra-centrifugation at 100,000 × *g* for 90 min at 4°C. The pellet was retained and re-suspended in buffer (1 mM EDTA, 10 mM Tris containing 0.33%vol 16% SDS) prior to further centrifugation for 45 min at 3,000 × *g*. Total protein was extracted by homogenization in Triton-X-100 extraction buffer (1% Triton-X-100, 150 mM NaCl, 50 mM of Tris-HCl, with a protease inhibitor cocktail) and centrifugation for 20 min at 800 × *g*. Protein concentration was determined for equine muscle extracts and total protein myocyte lysates in triplicate, using the bicinchoninic acid (BCA) protein assay kit (Pierce, IL, USA) with bovine serum albumin standards (intra-assay CV = 1.9%). Absorbance at 562 nm was measured on a microplate reader (Biotek, VT, USA).

Protein content of target proteins was analyzed in total lysates [SOCS3, TLR4 (myocytes), TNF-α, and IL-6] and crude membrane fractions (TLR4 and GLUTs) by quantitative Western blotting, as previously described (22, 24). Briefly, equal amounts of protein (15–25 μg) were resolved on an 8% (TLR4 and GLUTs) or 12% (IL-6, TNF-α, and SOCS3) SDS polyacrylamide gel and then electrophoretically transferred to a polyvinylidene fluoride membrane (Millipore, MA, USA) with subsequent immunoblotting. Membranes were probed with optimally diluted primary antibodies (GLUT4; 1:750, GLUT1; 1:500, GLUT8; 1:500, GLUT12; 1:500, TLR4; 1:200, IL-6; 1:500, TNF-α 1:1000, and SOCS3; 1:1000) either at room temperature for 1 h (GLUT4) or at 4°C for 16 h followed by incubation with an appropriate secondary antibody (1 h at room temperature) conjugated to horseradish peroxidase. Primary antibodies were purchased from Santa Cruz Biotechnology (GLUT1, GLUT8, and TLR4), Abcam (GLUT12, TNF-α, and SOCS3), R&D Systems (IL-6), and AbD Serotec (GLUT4). Protein content was assessed by enhanced chemiluminescence reaction (KPL, MD, USA) and quantified using a Gel-Pro Analyzer blot scanning and analysis system (Media Cybernetics, MD, USA). The mouse monoclonal TLR4 antibody had a close protein sequence homology with *Equus caballus* (98%), and thus, was used for both rat and equine preparations. The TNF-α and IL-6 antibodies were equine-specific. All these antibodies have been previously validated for use in horses (25). In addition, antibodies were validated against a positive control and equal protein loading was confirmed by measuring calsequestrin (1:2500, Thermo Scientific) protein expression following immunoblotting.

### Statistical analyses

The effect of *in vitro* treatment on myocyte TLR4 protein content was compared with a one-way ANOVA and Dunnett’s *post hoc* test. Clinical parameters and GLUT content were compared between p-EHC and control horse groups with a *t*-test. TLR4 and cytokine data did not satisfy requirements for normal distribution (Shapiro–Wilk) so were analyzed with non-parametric analyses (Mann–Whitney). Serum insulin was correlated with TLR4 protein content using Pearson’s correlation test. All data were expressed as mean ± standard error (SE) and significance was accepted at *p* < 0.05. Statistical analyses were performed using SigmaPlot v. 12.3.

## Results

### Effect of in vitro supraphysiologic insulin on cardiac myocyte TLR4 expression

**Figure 1:**
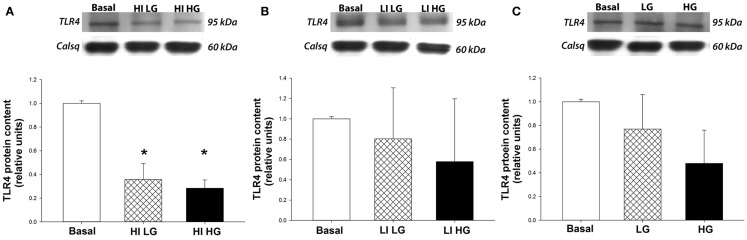
**High concentrations of insulin decrease TLR4 protein expression in isolated cardiac myocytes**. Top panels: representative Western blot of TLR4 protein expression from total lysates, with calsequestrin as a loading control. Bottom panels: mean ± SE of TLR4 protein expression (*n* = 5/group). *p < 0.05 vs. basal conditions. **(A)** Cardiac myocytes isolated from healthy rats show a decrease in TLR4 protein expression after incubation with insulin at high concentrations (HI, 500 μ IU/mL) in both physiological (LG, 5 mmoL/L) and high (HG, 25 mmoL/L) concentrations of glucose, compared to basal conditions. **(B)** TLR4 protein expression is not decreased in isolated myocytes following incubation with a physiological concentration of insulin (LI, 100 μ IU/mL) in both physiological (LG, 5 mmoL/L) and high (HG, 25 mmoL/L) concentrations of glucose, compared to basal conditions. **(C)** Incubation of isolated myocytes with glucose does not affect TLR4 protein expression in the absence of insulin.

Isolated rat cardiac myocytes exhibited a decrease (*p* < 0.05) in TLR4 protein expression by 65 and 69% when incubated with a supraphysiological concentration of insulin (500 μ IU/mL) in the presence of both physiological (5 mM) or high (25 mM) concentrations of glucose, respectively, compared to basal conditions (Figure 1A). In contrast, incubation of isolated myocytes in physiological concentration of insulin (100 μ IU/mL) did not result in a change in TLR4 protein expression, compared to basal conditions, in either physiological or high concentrations of glucose (Figure 1B). Further, incubation of myocytes in both physiological (5 mM) or high (25 mM) concentrations of glucose, in the absence of insulin, did not significantly alter TLR4 expression compared to basal conditions (Figure 1C).

### Effect of prolonged insulin infusion on cardiac TLR4 expression

To further investigate the role of insulin on TLR4 expression and signaling, prolonged insulin infusion for ~46 h was performed in a large animal model. As expected, the euglycemic hyperinsulinemic clamp induced a marked prolonged exogenous hyperinsulinemia, without a change in blood glucose concentration (Table 1). In addition, prolonged hyperinsulinemia did not affect heart rate and post-mortem examination did not reveal any gross pathological abnormalities of the myocardium. Matched control horses did not experience any changes in insulin or glucose concentrations during infusion of the balanced electrolyte solution (Table 1).

**Table 1:** **Mean ± SE heart rate, serum insulin, and blood glucose concentration before and after intravenous insulin infusion**.

Parameter	Heart rate (beats/min)	Serum insulin (μ IU/mL)	Blood glucose (mmoL/L)
Control group: baseline	38 ± 3.46	13.36 ± 2.7	5.16 ± 0.68
Control group: final	43 ± 6.19	10.05 ± 1.16	5.90 ± 0.68
Treated group: baseline	36 ± 3.26	22.2 ± 11.3	5.78 ± 0.39
Treated group: final	44 ± 4.32	1179 ± 228*	5.05 ± 0.56

Heart rate did not differ between horses treated with a prolonged, euglycemic, hyperinsulinemic clamp (p-EHC), and control horses treated with a balanced electrolyte solution either before or at the conclusion of the infusion period (i.e., 46 ± 2.3 h). Serum insulin concentration was markedly increased (*p < 0.05) from baseline levels as a consequence of treatment with a p-EHC, but not with a balanced electrolyte solution (controls). However, blood glucose concentration did not differ between groups or as a consequence of treatment.

**Figure 2:**
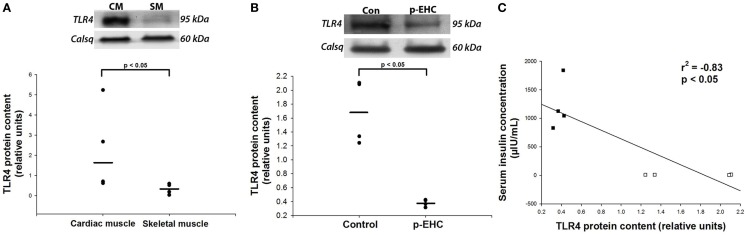
**Prolonged hyperinsulinemia down-regulates cardiac TLR4 expression**. **(A,B)** Representative Western blot of TLR4 protein expression from crude membrane fractions; calsequestrin (Calsq) was used as a loading control. **(A)** TLR4 protein expression is greater (*p* < 0.05) in the heart than in skeletal muscle in healthy horses (*n* = 4). **(B)** TLR4 protein expression is decreased (*p* < 0.05) in horses treated with a prolonged, euglycemic, hyperinsulinemic clamp (p-EHC), compared with control horses. **(C)** Serum insulin concentration negatively correlates (Rsqr = −0.83) with myocardial TLR4 protein content in horses treated with a p-EHC (■) and control horses (□).

Since cardiac TLR4 protein expression has not been characterized in this species, we first quantified TLR4 expression across striated muscle of healthy horses. Given that TLR4 is overwhelmingly expressed on the plasma membrane and Golgi apparatus, we specifically extracted cell membrane-rich fractions of tissue lysates for analysis (26). We found that the myocardium expressed significantly more TLR4 protein than the skeletal muscle (Figure 2A). In addition, myocardial protein expression of TLR4 was decreased (*p* < 0.05) by 77% in crude membrane fractions from horses treated with a p-EHC, compared to the control group (Figure 2B). Furthermore, there was a significant (*p* < 0.05) negative correlation between serum insulin concentration and TLR4 protein expression, suggesting that insulin negatively regulates TLR4 in the myocardium (Figure 2C).

### Cardiac TLR4 signaling and GLUT expression during hyperinsulinemia

**Figure 3:**
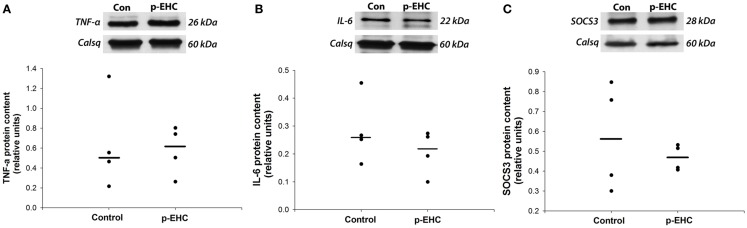
**Markers of TLR4 signaling are not affected by hyperinsulinemia in horses**. Top panels: representative Western blots with calsequestrin (Calsq) as a loading control. Bottom panels: median myocardial protein expression of the pro-inflammatory cytokines tumor necrosis factor-alpha (TNF-α) **(A)** and interleukin-6 (IL-6) **(B)** do not differ between horses treated with a prolonged, euglycemic, hyperinsulinemic clamp (p-EHC), compared with control horses. Similarly, protein content of suppressor of cytokine signaling 3 (SOCS3) is not affected in horses treated with a p-EHC, compared with control horses **(C)**.

**Figure 4:**
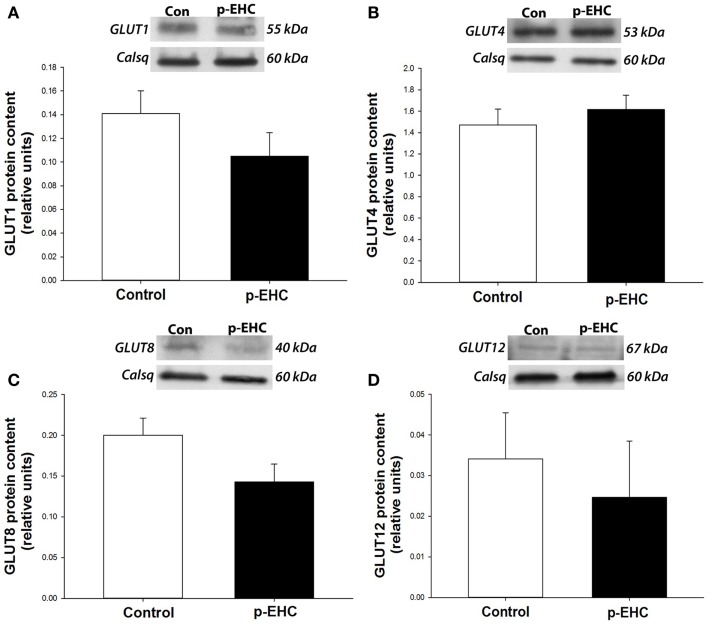
**Hyperinsulinemia and concomitant down-regulation of TLR4 does not alter glucose transporter (GLUT) protein expression**. Top panels: representative Western blots of GLUT expression from cardiac crude membrane fractions; calsequestrin (Calsq) was used as a loading control. Bottom panels: mean ± SE protein expression of GLUTs 1 **(A)**, 4 **(B)**, 8 **(C)**, and 12 **(D)** do not differ in the ventricular myocardium of horses treated with a prolonged, euglycemic, hyperinsulinemic clamp (p-EHC), compared with control horses.

To further investigate potential downstream effects of reduced TLR4 expression in the myocardium during *in vivo* hyperinsulinemia, protein expression of the TLR4 signaling markers (SOCS3, TNF-α, and IL-6) and GLUT (1, 4, 8, 12) was examined. Concomitant to the down-regulation of myocardial TLR4 protein content, protein expression of TNF-α, IL-6, and SOCS3 did not differ between control and p-EHC-treated animals (Figure 3). Further, treatment with a p-EHC did not alter myocardial protein expression of basal GLUT1, insulin-sensitive GLUT4, or the novel GLUTs (8 and 12) in membrane-enriched fractions, when compared to controls (Figure 4).

## Discussion

It is well established that increased inflammation is a major pathogenic factor in the development of many chronic diseases, including diabetes and cardiovascular diseases (11, 27–29). Our study demonstrated that acute and prolonged insulin exposure has a potent suppressive effect on the expression of TLR4, a key regulator of innate immune and inflammatory responses, in the mammalian heart, and thus could be a promising pharmacological agent in the treatment of cardiometabolic diseases.

Studies on TLR signaling in insulin-sensitive tissues have overwhelmingly reported an increase in TLR4 expression during metabolic dysfunction (13, 30). Activation of TLR4 signaling by increased circulating FFAs during obesity-related diseases has been suggested to be a key contributor to this up-regulation of TLR4 signaling (30, 31). However, examinations of the effect of insulin on TLR4 signaling in non-obese individuals, where circulating FFAs are not increased, are far less common. A recent study showed that short-term insulin infusions administered to type 1 diabetic patients decreased TLR expression in mononuclear cells (14). Results from the current study have also shown decreased TLR4 protein expression in a cardiac crude membrane extract during conditions of prolonged (46 h) insulin infusion in healthy equine subjects. In addition, acute incubation with supraphysiologic concentration of insulin decreased TLR4 expression in total lysate from isolated murine cardiac myocytes. Therefore, our findings suggested that prolonged hyperinsulinemia induces impairment in TLR4 protein expression and/or translocation to the plasma membrane. This premise is further supported by reports that IGF-1, which triggers the same intracellular pathways as insulin following receptor binding, induced suppression of TLR4 signaling in skeletal muscle (32). In addition, a study demonstrating suppression of TLR activation by phosphoinositide 3-kinase (a key enzyme of the downstream insulin signaling pathway) indicates a potential mechanism by which insulin may down-regulate TLR signaling (33). However, further investigation of this pathway in the non-immune cells of the heart is required. Since excessive inflammation can be harmful, negative regulation of TLR4 signaling by insulin may be a protective mechanism to prevent inappropriate innate immune responses in the heart. However, whether hyperinsulinemia can down-regulate the activation of TLR4 during obesity-related diseases require further investigation.

Although metabolic diseases have been overwhelmingly associated with an inflammatory phenotype (11, 27, 29), our results suggest that hyperinsulinemia does not directly increase pro-inflammatory cytokine production (i.e., IL-6 and TNF-α) in the healthy heart, suggesting that other factors, such as obesity, play a pathogenic role during the inflammatory response. Indeed, many of the animal models used to study cardiac inflammation and metabolic dysfunction *in vivo* are complicated by obesity. Our model of prolonged equine hyperinsulinemia is unique in that it facilitates investigation of the effects of hyperinsulinemia in insulin-sensitive, non-obese individuals from a species that is prone to the development of metabolic syndrome, but not diabetes mellitus or cardiovascular diseases (34, 35). The use of prolonged clamping techniques in this species has added valuable information to the field of metabolic research as the EHC is limited to shorter periods (3–8 h) in rodents and humans (36, 37). The ability to prolong an EHC to 46 h in the horse enables the investigation of longer term, marked hyperinsulinemia in a controlled manner. Investigations using this model have yielded important progress in our understanding of equine metabolic disease (38, 39). However, the impact of marked hyperinsulinemia on the healthy heart, which was demonstrated not to be insulin-resistant at the time of examination, by a lack of down-regulation of GLUT4 expression, is reported here for the first time. In addition, the presence of significantly more TLR4 in the myocardium than in skeletal muscle of healthy horses in the current study would suggest that TLR signaling is highly physiologically relevant to the heart. This is supported by recent studies, which have provided strong evidence that TLR4 signaling not only mediates myocardial inflammation and ischemic injury but also contributes to cardiac dysfunction during metabolic disease (40, 41). Studies have also shown that defective insulin dynamics are thought to be among the earliest pathophysiological changes in the diabetic myocardium, preceding structural and functional changes, and thus, are of major importance in the onset and progression of cardiovascular dysfunction during metabolic diseases (42).

The down-regulation of TLR4 combined with the lack of increased production of pro-inflammatory cytokines (i.e., IL-6 and TNF-α) and SOCS3 following insulin infusion in the current study suggested that hyperinsulinemia exerts an anti-inflammatory effect on the heart in non-obese individuals. Similarly, the anti-inflammatory effect of insulin on both immune and non-immune cells has been demonstrated (43, 44). These anti-inflammatory effects have also been shown to occur *in vivo*, providing strong evidence that insulin has the potential to be cardioprotective (45–47). Indeed, insulin infusion has long been known to be effective in the treatment of patients with heart failure or undergoing coronary bypass surgery, although its cardioprotective mechanisms have not been elucidated (48). It has been suggested that the therapeutic effect of insulin could be related to tighter glycemic control, since it has been suggested that hyperglycemia stimulates inflammatory pathways in immune cells (14, 49). However, in the present study, we reported that high concentrations of insulin, but not of glucose, affected TLR4 expression in isolated cardiac myocytes, suggesting a new mechanism for the cardioprotective effect of insulin. The potential for insulin to be cardioprotective via reduced TLR4-mediated inflammation is also highlighted by studies that demonstrated that inhibition of TLR4 signaling preserves cardiac structure and function in non-obese type 1 diabetic mice (41). Further, studies on adipose tissue using mouse models with selective post-receptor, loss-of function mutations in TLR4 have demonstrated that TLR4 deficiency is protective against the deleterious inflammatory effects associated with obesity and metabolic disease (50–53). Clearly, given the likelihood for hyperglycemia to play a central role in cardiac malfunction during diabetes, the impact of hyperinsulinemia on TLR4 expression during metabolic diseases, such as in pre-diabetic and diabetic heart disease, requires investigation (14, 54, 55).

The mechanism underlying how aberrant TLR signaling contributes to cardiac IR is proposed to be related to impaired glucose uptake into myocytes (56, 57). Glucose utilization is approximately four times greater in the myocardium than in either skeletal muscle or adipose tissue, despite its ability to utilize other metabolic substrates; thus, maintenance of normal cardiac muscle function relies on efficient glucose uptake and utilization (42). The heart relies principally on GLUT1 and GLUT4 (basal and insulin-sensitive isoforms, respectively), although other novel isoforms, including GLUT12 and GLUT8, have recently been shown to play important roles in cardiac function, indicating the importance of investigating all major cardiac GLUT isoforms in studies of cardiac glucose metabolism (58, 59). However, there is considerable debate on the function of these novel GLUT isoforms and as to whether they are basal or insulin-dependent transporters, although GLUT12 was recently characterized as a non-insulin stimulated GLUT in the myocardium (58). This finding is in agreement with the results of the current study that demonstrated that prolonged insulin infusion did not affect the expression of basal GLUTs in the myocardium, including GLUT1 and 12. GLUT4, which accounts for at least 60% of the cardiac GLUTs, plays a crucial role in whole-body glucose homeostasis (60, 61). Given that activation of TLR4 may impair cardiac glucose metabolism, it has been postulated that decreased TLR4 expression may improve cardiac insulin sensitivity. The failure of membrane-rich GLUT4 expression to respond to reduced TLR4 expression secondary to hyperinsulinemia in the current study could suggest that TLR4 signaling does not primarily regulate glucose transport in the myocardium in contrast to the skeletal muscle (62). Indeed, the regulation of glucose transport has been shown to be tissue specific. Since the heart contracts constantly, it has been suggested that calcium/contraction pathways are also major regulators of GLUT4 translocation in the myocardium (63). Therefore, the regulation of cardiac glucose metabolism by insulin-independent pathway and the relative function of cardiac GLUTs requires further investigation. In addition, glucose transport could have also been regulated by other factors (e.g., transcriptional) that may not have been detected by the end time point of the prolonged clamp (i.e., 46 h). Therefore, the relationship between TLR4 signaling and GLUT regulation in the heart is intriguing and further investigation may not only uncover new mechanisms for cardiac IR but also allow identification of novel therapeutic targets for diabetic cardiomyopathy.

Overall, the current study has presented the novel finding that negative regulation of TLR4 expression exists in the heart during hyperinsulinemia using an integrative approach, including a unique, large animal model. This study shows the potential for insulin to be cardioprotective via reduced TLR4-mediated inflammation and this finding requires further investigation in human diabetic patients. Inactivation of aberrant TLR4 function during metabolic disease may provide novel therapeutic targets for the treatment or prevention of cardiovascular diseases in individuals with diabetes.

## Conflict of Interest Statement

The authors declare that the research was conducted in the absence of any commercial or financial relationships that could be construed as a potential conflict of interest.
